# Enhanced Intestinal Motility during Oral Glucose Tolerance Test after Laparoscopic Sleeve Gastrectomy: Preliminary Results Using Cine Magnetic Resonance Imaging

**DOI:** 10.1371/journal.pone.0065739

**Published:** 2013-06-18

**Authors:** Vo Nguyen Trung, Hiroshi Yamamoto, Akira Furukawa, Tsuyoshi Yamaguchi, Satoshi Murata, Masahiro Yoshimura, Yoko Murakami, Shigetaka Sato, Hideji Otani, Satoshi Ugi, Katsutaro Morino, Hiroshi Maegawa, Tohru Tani

**Affiliations:** 1 Department of Surgery, Shiga University of Medical Science, Seta-Tsukinowa-cho, Otsu, Shiga, Japan; 2 Department of Radiology, Shiga University of Medical Science, Seta-Tsukinowa-cho, Otsu, Shiga, Japan; 3 Department of Medicine Shiga University of Medical Science, Seta-Tsukinowa-cho, Otsu, Shiga, Japan; Hirosaki University Graduate School of Medicine, Japan

## Abstract

**Background:**

Enhanced secretion of glucagon-like peptide-1 (GLP-1) has been suggested as a possible mechanism underlying the improvement in type 2 diabetes mellitus (T2DM) after laparoscopic sleeve gastrectomy (LSG). However, the reason for enhanced GLP-1 secretion during glucose challenge after LSG remains unclear because LSG does not include intestinal bypass. In this study, we focused on the effects of LSG on GLP-1 secretion and intestinal motility during the oral glucose tolerance test (OGTT) using cine magnetic resonance imaging (MRI) before and 3 months after LSG.

**Methods:**

LSG was performed in 12 obese patients with a body mass index >35 kg/m^2^. Six patients had T2DM. OGTT was performed before and 3 months after the surgery. Body weight, hemoglobin A1c (HbA1c), and GLP-1 levels during OGTT were examined, and intestinal motility during OGTT was assessed using cine MRI.

**Results:**

Body weight was significantly decreased after surgery in all the cases. HbA1c was markedly decreased in all the diabetic subjects. In all cases, GLP-1 secretion during OGTT was enhanced and cine MRI showed markedly increased intestinal motility at 15 and 30 min during OGTT after LSG.

**Conclusions:**

LSG leads to accelerated intestinal motility and reduced intestinal transit time, which may be involved in the mechanism underlying enhanced GLP-1 secretion during OGTT after LSG.

## Introduction

Morbid obesity is currently a worldwide health problem because it promotes the development of various diseases, including cardiovascular disease and type 2 diabetes mellitus (T2DM), which considerably increase mortality. However, current therapies, such as diet, exercise, lifestyle modification, and medication, seem to be insufficient for treating morbid obesity.

There is strong evidence that bariatric surgery can cure not only obesity but also its comorbidities. Laparoscopic sleeve gastrectomy (LSG) is typically performed before biliopancreatic diversion in the treatment of morbid obesity for high-risk patients, but LSG has recently been applied as a single-stage procedure because of its technical simplicity, remarkable postoperative weight loss, and T2DM remission [1]–[3].

However, the mechanism underlying the improvement in T2DM after LSG has not been elucidated until now. Enhanced secretion of glucagon-like peptide-1 (GLP-1) has been suggested to be a mechanism underlying the improvement in T2DM after LSG [4]–[7]. However, the reason for enhanced GLP-1 release during glucose challenge after LSG remains unclear because LSG does not include intestinal bypass as part of the technique.

A previous study with scintigraphy [8] demonstrated that gastric emptying accelerates after LSG, which may promote GLP-1 release through a neuroendocrine loop to the distal small intestine [9], [10], whereas another study did not demonstrate the same [11]. Because intestinal L cells, which secrete GLP-1, are mainly located in the distal intestine [12], studies investigating the effect of LSG on intestinal motility, including that of the distal intestine, are necessary. However, data on intestinal motility after LSG have been reported only using the scintigraphy method [13], [14]. Cine magnetic resonance imaging (MRI) is a new imaging technology that provides direct visualization of intestinal contraction and peristalsis [15]. We conducted the present study to investigate the effects of LSG on GLP-1 secretion and intestinal motility during the oral glucose tolerance test (OGTT) using cine MRI before and 3 months after LSG.

## Materials and Methods

### Patients

Twelve obese patients with a body mass index (BMI) >35 kg/m^2^ were recruited into the study. Among these, 6 patients had T2DM and 2 diabetic patients had hemoglobin A1c (HbA1c) levels >7.8%. Patients were eligible to participate in the study if they were between 20 and 65 years of age, and had a BMI between 30 and 35 kg/m^2^ with T2DM. Candidates were excluded if they had type 1 diabetes, severe diabetic complications, or a contradiction for either surgery. We also excluded subjects with a history of gastrointestinal motility disorders or inflammatory bowel disease. In addition to any assessments required for inclusion, each participant was assessed by a multidisciplinary team. The Ethics Review Committee of Shiga University of Medical Science (Shiga, Japan) approved all protocols described in this study, and all participants provided written informed consent.

### Procedure

LSG was performed with the patient in a supine position using a standard 5-port laparoscopic technique with a 45-Fr gastric tube to calibrate the sleeve, and dissection of the greater curvature began approximately 5–6 cm from the pylorus, as described previously [16].

On the first day after surgery, the patient was administered a clear liquid diet, which progressed to a complete liquid diet for 2 weeks, followed by a soft diet for 1 week, eventually advancing to a regular diet.

### Measurement of GLP-1_7–36_ during OGTT

OGTT was performed 1 week before and 3 months after the surgery. GLP-1_7–36_ levels during OGTT were measured using commercially available enzyme-linked immunosorbent assay kits (Linco Research Inc., St. Charles, MO, USA).

### MRI Protocol

MRI examinations were performed for each patient 1 week before and 3 months after the surgery. After 8 h of fasting, MRI was performed before as well as 15 and 30 min after oral intake of 225 mL of fluid containing 75 g of glucose. Imaging was performed as reported previously [15], using a 1.5T MR scanner (Signa HDxt 1.5T; GE Healthcare, Milwaukee, WI, USA) with an 8-channel body array coil. Before real cine MRI, coronal images of the entire abdomen were obtained to determine the optimal image plane covering the maximum length of the small bowel loops. A serial coronal scan consisting of 50 images was obtained at the selected plane with the patient in a supine position in 25 s during breath holding. The steady-state free precession sequence (FIESTA sequence: TR, 3.4 ms; TE, 1.2 ms; flip angle, 75°; slice thickness, 10 mm; matrix, 256 × 256; field of view, 450 mm) was used for imaging. Intestinal motility was assessed by cine MRI on a monitor using a “cine-loop” display [15].

Based on the cine MRI, 2 bowel segments, one located in the left upper quadrant as representative of the jejunal loops and the other located in the right lower quadrant as representative of the ileal loops, were chosen for assessment of contraction. In this process, bowel loops with a degree of distension similar to the rest of the loops in the same quadrant as well as remaining in the image plane during the sequential imaging without displacement out of the image plane were chosen for assessment. Frequencies of bowel contractions were counted visually on a monitor using cine MRI. Arrival of the orally administered fluid to the jejunum, ileum, and terminal ileum was assessed within each sequence (15 and 30 min after glucose intake) by the presence or absence of bowel distension and high signal fluid. The presence or absence of distension of the jejunal and ileal loops was also judged, and contraction frequencies were compared between distended and collapsed bowel loops after surgery.

### Statistical Analysis

We analyzed the data using SPSS version 17.0 software (SPSS Inc., Chicago, IL, USA) and the paired sample *t*-test. Data are presented as mean ± standard deviation. Area under the curves (AUCs) of GLP-1_7–36_ during OGTT was calculated by trapezoidal integration. A *p* value <0.05 was considered statistically significant.

## Results

### Effect on Weight Loss and HbA1c

The percentage of excess weight loss (%EWL) at 3 months after the surgery was 48% ±22% (Table S1). All 6 diabetic patients discontinued all diabetic medications immediately after the surgery, and their HbA1c levels significantly decreased (Table S1).

### Effect on GLP-1_7–36_ Secretion during OGTT

In both the nondiabetic and diabetic patients, GLP-1_7–36_ secretion during OGTT was significantly enhanced. AUC of GLP-1_7–36_ was significantly higher after LSG than before LSG (Figure S1).

### Intestinal Motility

Cine MRI scans before and 3 months after LSG were obtained in 9 of the 12 patients because of 3 patients refused examination, including 2 diabetic patients and 1 nondiabetic patient. Cine MRI was tolerated well in all 9 patients and provided sufficient quality of cine images to analyze bowel contractions and the state of small bowel transit. There was no significant difference in mean frequencies of contractions of the jejunum and ileum prior to glucose intake between before and after LSG. However, their contractions significantly increased at 15 and 30 min after glucose intake after LSG compared with those before LSG (Table S2).

The percentage of patients whose glucose fluid reached the jejunum, ileum, and ileum terminal at 15 and 30 min after fluid intake was markedly increased after LSG (Table S3). Before LSG, 33%, 11%, and 0% of patients showed the presence of fluid in the jejunum, ileum, and ileum terminal at 15 min after fluid intake, respectively. After LSG, 100%, 89%, and 89% of patients showed the presence of fluid in the jejunum, ileum, and ileum terminal, respectively. Moreover, all patients showed the presence of fluid in the jejunum, ileum, and ileum terminal at 30 min after fluid intake following LSG compared with 41%, 33%, and 22% before LSG, respectively (Table S3).

In addition, the mean frequency of contractions of fluid-distended jejunum and ileum loops (6.1/min and 7.4/min, respectively) was significantly higher than that of contractions of collapsed jejunum and ileum loops (0.5/min and 1.4/min, respectively) (Table S4).

We included video clips of 4 representative patients as a demonstration of changes in cine MRI after LSG (Table S5). The results of the remaining patients were the same.

## Discussion

Our study offers novel insights into the effects of LSG on intestinal motility using the novel cine MRI method. In summary, we demonstrated that LSG enhances GLP-1_7–36_ secretion and accelerates intestinal motility and propagation of the test fluid during OGTT.

Previous studies have shown that LSG leads to improvement in glucose tolerance, which may be explained by the decrease in insulin resistance due to weight loss [17], [18] and by the increase in insulin secretion due to enhanced GLP-1 secretion [6], [7], [19], [20]. GLP-1 is important for remission of diabetes but may not have a major role in sleeve gastrectomy, since Wilson-Perez HE showed that sleeve gastrectomy is still effective in mice lacking the GLP-1 receptor [21]. In the present study, the changes in GLP-1 secretion and intestinal motility after LSG rather than GLP-1 and its effects on the resolution of diabetes after LSG was the main focus.

To date, the reason for enhanced GLP-1 secretion after LSG still remains unclear because LSG does not include intestinal bypass. Patel RT et al. have recently depicted the role of the duodenum in promoting high levels of GLP-1 following sleeve gastrectomy [20]. In addition, several studies postulated that some implications of the accelerated gastric emptying or intestinal motility could be involved in the mechanism of increased GLP-1 secretion after LSG [8], [13], [14], [22]; however, most studies investigating gastrointestinal motility after LSG employed only scintigraphy.

Our main scope was to evaluate postoperative alterations in small intestinal motility, an area much more obscure than the stomach. There are several ways to monitor and assess small bowel motility function, such as transit time analysis [23]–[25], manometry [26]–[28], impedancometry, and tensiometry [29], [30]; however, each method has advantages and limitations, and there is no single established noninvasive method for clinical use [15]. Conventional x-ray methods such as enterography and enteroclysis may also be used to examine the small bowel [31]; however, it is not suitable for long or repetitive examinations because of radiation exposure. With recent advances in technology, MRI has been used for diagnosing various gastrointestinal disorders [32]–[35], and preliminary trials have reported the potential of MRI to monitor and assess bowel motility [15], [36]–[41]. Cine MRI using subsecond ultrafast scanning sequences presents a noninvasive method to assess bowel motility function because of the high temporal, spatial, and contrast resolution [15], [38], [41]–[43]. The present study is the first to investigate the potential of MRI using a steady-state free precession sequence to assess bowel motility function after LSG. The steady-state free precession sequence is a fast imaging sequence providing motion-free images with “T2-like” contrast, which has typically been used in cardiac imaging [15], [44], [45]. With technical development, steady-state free precession sequences can now provide motion-free images with high spatial resolution and temporal resolution slightly less than 0.5 s per image, which is suitable to monitor small bowel motility. Coronal imaging in the prone position is used to separate the bowel loops and to reduce the displacement of the intestinal structures in the imaging section. Consequently, cine-dynamic coronal MRI with a section thickness of 10 mm covers a large portion of the small bowel loops [15].

Thus, cine MRI can enable visualization of movement of the entire intestine in real time before and after glucose intake. Using this novel method, we showed that intestinal motility was markedly accelerated and small bowel transit time was reduced after glucose intake in all patients following LSG. This acceleration of contraction was concurrently observed with faster arrival of the intake fluid and intestinal distension. The arrival of the glucose fluid might have changed the patterns of bowel contraction from a fasting pattern to a postprandial pattern. As demonstrated by Wakamiya et al. [15], distended loops contract more frequently than collapsed loops, as was also observed in the present study.

Gastrointestinal motility is an integrated process that includes myoelectrical and contractile activities, tone, compliance, and transit [46]. These different aspects of motility are regulated by complex mechanisms involving the central nervous system, local neuronal control, and circulating neurohormonal substances [14]. Theoretically, LSG may affect gastric emptying by several mechanisms, such as removal of the fundus with its receptive and propulsive abilities, altered compliance and contractility of the narrow nondistensible sleeve [47], removal of the gastric pacemaker area from the body of the stomach, and compromise of the action of the antral pump if part of the antrum is resected. However, changes in small bowel motility are even more difficult to explain in relation to known physiological mechanisms [14]. Shortening of gastric emptying time and early arrival of the oral intake fluid to the small bowel may be important factors that stimulate intestine contractions and consequently accelerate bowel propagation, thereby leading to the early arrival of the glucose fluid at the ileum terminal. However, Melissas et al. [14] demonstrated that small bowel transit is accelerated independently after LSG and not just because of faster gastric emptying. In addition, it may seem paradoxical that increased gastrointestinal motility stimulates GLP-1 release, which has been suggested to inhibit gastrointestinal motility [12], [48]; however, this discrepancy can be explained in several ways. For example, Salehi et al. [49] distinguished between GLP-1 actions that are physiological, such as the regulation of islet hormone secretion, and others, such as gastrointestinal motility, which may only be relevant at pharmacological GLP-1 levels. In addition, vagal afferent nerves have been shown to mediate the inhibitory action of GLP-1 on gastrointestinal motility; therefore, partly compromised vagal fibers in the stomach after LSG can attenuate the effects of GLP-1 on gastrointestinal motility [50]. On the other hand, reduction in other hormone levels, including leptin and amylin, which has been observed after LSG, can be associated with enhanced gastrointestinal motility [51]–[54]. Finally, it is unclear if gastric emptying and intestinal motility would be even higher if not for the increased release of GLP-1 in LSG patients [1]; thus, future studies designed to assess these parameters are needed.

In agreement with previous studies [13], [14], we postulated that the acceleration in intestinal motility after LSG by itself may have enhanced the exposure of ileal L cells to the mixture of glucose fluid and digestive juices and subsequently increased GLP-1 release.

We acknowledged several limitations in the present study. As a first pilot study, we only enrolled 12 patients, and of these, only 9 underwent cine MRI. In addition, our reliance on liquid test meals appears to be the most serious limitation; thus, evaluation of gastrointestinal motility after ingestion of a solid test meal is required before our results can be generalized.

Nevertheless, for the first time, we clearly demonstrated that intestinal motility was markedly accelerated and bowel transit time reduced after LSG using a novel method.

In conclusion, LSG leads to accelerated intestinal motility and reduced intestinal transit time, which may be involved in the mechanism underlying enhanced GLP-1 secretion during OGTT after LSG. However, the exact mechanisms by which LSG affects intestinal motility remain unknown; therefore, further studies are needed to clarify this issue.

## Supporting Information

Figure S1 **GLP-1 levels during OGTT before and 3 months after surgery.** A: Nondiabetic patients. B: Diabetic patients. Data are presented as mean ± standard deviation. *p<0.05 and **p<0.01. GLP-1: glucagon-like peptide-1; OGTT: oral glucose tolerance test; AUC: area under the curve.
(TIF)

Table S1 **Changes in body weight and HbA1C 3 months after surgery.** T2DM: type 2 diabetes mellitus; EWL: excess weight loss; HbA1C: hemoglobin A1C. Data were shown as mean ± standard deviation
(DOC)

Table S2 **Changes in contraction of the jejunum and ileum during OGTT 3 months after surgery.** OGTT: oral glucose tolerance test. Data are presented as mean ± standard deviation.
(DOC)

Table S3 **Changes in frequency of the presence of glucose fluid in the jejunum, ileum, and ileum terminal during OGTT 3 months after surgery.** OGTT: oral glucose tolerance test.
(DOC)

Table S4 **Differences in contraction frequencies between collapsed and fluid-distended bowel loops during OGTT 3 months after surgery.** OGTT: oral glucose tolerance test. Data are presented as mean ± standard deviation.
(DOC)

Table S5 **Representative results of intestinal motility during OGTT in 4 patients assessed by cine MRI.** T2DM: type 2 diabetes mellitus; OGTT: oral glucose tolerance test; MRI: magnetic resonance imaging.
(DOC)

Video S1 **Assessment of intestinal motility during OGTT before and 3 months after surgery in diabetic patient #1 by cine MRI.** Video S1A and video S1B: at 0 and 15 min during OGTT before surgery, respectively. Video S1C and video S1D: at 0 and 15 min during OGTT after surgery, respectively. OGTT: oral glucose tolerance test; MRI: magnetic resonance imaging.
(ZIP)

Video S2 **Assessment of intestinal motility during OGTT before and 3 months after surgery in diabetic patient #2 by cine MRI.** Video S2A and video S2B: at 0 and 15 min during OGTT before surgery, respectively. Video S2C and video S2D: at 0 and 15 min during OGTT after surgery, respectively. OGTT: oral glucose tolerance test; MRI: magnetic resonance imaging.
(ZIP)

Video S3 **Assessment of intestinal motility during OGTT before and 3 months after surgery in nondiabetic patient #3 by cine MRI.** Video S3A and video S3B: at 0 and 15 min during OGTT before surgery, respectively. Video S3C and video S3D: at 0 and 15 min during OGTT after surgery, respectively. OGTT: oral glucose tolerance test; MRI: magnetic resonance imaging.
(ZIP)

Video S4 **Assessment of intestinal motility during OGTT before and 3 months after surgery in nondiabetic patient #4 by cine MRI.** Video S4A and video S4B: at 0 and 15 min during OGTT before surgery, respectively. Video S4C and video S4D: at 0 and 15 min during OGTT after surgery, respectively. OGTT: oral glucose tolerance test; MRI: magnetic resonance imaging.
(ZIP)
