# Supplementary figures and images for: Enhanced Intestinal Motility during Oral Glucose Tolerance Test after Laparoscopic Sleeve Gastrectomy: Preliminary Results Using Cine Magnetic Resonance Imaging
Source: PLoS One. 2013 Jun 18;8(6):e65739. doi: 10.1371/journal.pone.0065739 (PMC3688799; doi:10.1371/journal.pone.0065739)

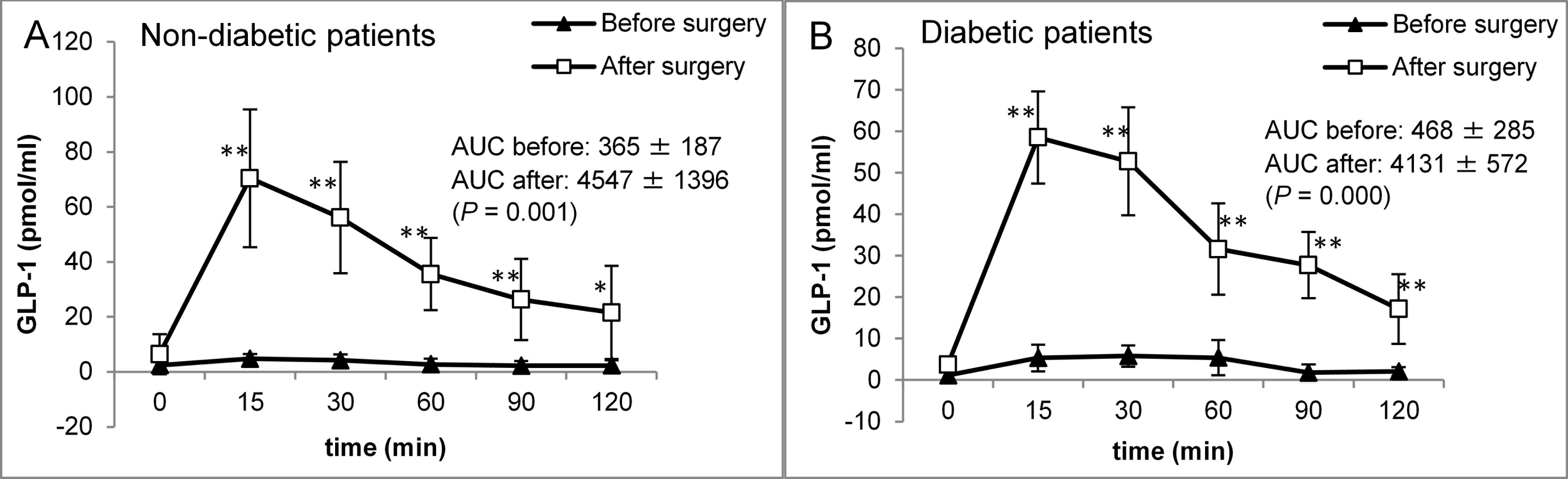

Supplement: Figure S1 — GLP-1 levels during OGTT before and 3 months after surgery. A: Nondiabetic patients. B: Diabetic patients. Data are presented as mean ± standard deviation. *p<0.05 and **p<0.01. GLP-1: glucagon-like peptide-1; OGTT: oral glucose tolerance test; AUC: area under the curve. (TIF) [file pone.0065739.s001.tif]
