# Supplementary material for: Enhanced Intestinal Motility during Oral Glucose Tolerance Test after Laparoscopic Sleeve Gastrectomy: Preliminary Results Using Cine Magnetic Resonance Imaging
Source: PLoS One. 2013 Jun 18;8(6):e65739. doi: 10.1371/journal.pone.0065739 (PMC3688799; doi:10.1371/journal.pone.0065739)
Supplement: Table S1 — Changes in body weight and HbA1C 3 months after surgery. T2DM: type 2 diabetes mellitus; EWL: excess weight loss; HbA1C: hemoglobin A1C. Data were shown as mean ± standard deviation (DOC) [file pone.0065739.s002.doc]

**Table S1. Changes in body weight and HbA1C 3 months after surgery**

| **Parameters** | **Patient** | **Before surgery** | **3 months after surgery** | ***p*** |
| --- | --- | --- | --- | --- |
| Body weight | All | 109.7 ± 26.4 | 89.5 ± 25.4 | 0.000 |
| EWL (%) | All |  | 48 ± 22 |  |
| HbA1C (%) | T2DM | 8.0 ± 1.4 | 6.0 ± 0.8 | 0.006 |
|  | Non-T2DM | 5.2 ± 0.4 | 5.1 ± 0.4 | 0.2 |

T2DM: type 2 diabetes mellitus; EWL: excess weight loss; HbA1C: hemoglobin A1C. Data were shown as mean ± standard deviation
