# Supplementary material for: Enhanced Intestinal Motility during Oral Glucose Tolerance Test after Laparoscopic Sleeve Gastrectomy: Preliminary Results Using Cine Magnetic Resonance Imaging
Source: PLoS One. 2013 Jun 18;8(6):e65739. doi: 10.1371/journal.pone.0065739 (PMC3688799; doi:10.1371/journal.pone.0065739)
Supplement: Table S2 — Changes in contraction of the jejunum and ileum during OGTT 3 months after surgery. OGTT: oral glucose tolerance test. Data are presented as mean ± standard deviation. (DOC) [file pone.0065739.s003.doc]

**Table S2. Changes in contraction of the jejunum and ileum during OGTT 3 months after surgery**

| **Parameters** | **Before surgery** | **3 months after surgery** | ***p*** |
| --- | --- | --- | --- |
| Contraction of the jejunum at 0 min (frequency/min) | 0.4 ± 0.8 | 0.5 ± 1.1 | 0.7 |
| Contraction of the jejunum at 15 min (frequency/min) | 2.4 ± 2.8 | 6.1 ± 0.7 | 0.01 |
| Contraction of the jejunum at 30 min (frequency/min) | 3.2 ± 2.0 | 5.7 ± 1.4 | 0.02 |
| Contraction of the ileum at 0 min (frequency/min) | 1.3 ± 1.7 | 1.2 ± 1.7 | 0.8 |
| Contraction of the ileum at 15 min (frequency/min) | 2.4 ± 2.7 | 7.0 ± 2.4 | 0.009 |
| Contraction of the ileum at 30 min (frequency/min) | 3.4 ± 2.7 | 7.4 ± 1.4 | 0.002 |

OGTT: oral glucose tolerance test. Data are presented as mean ± standard deviation.
