# Supplementary material for: Enhanced Intestinal Motility during Oral Glucose Tolerance Test after Laparoscopic Sleeve Gastrectomy: Preliminary Results Using Cine Magnetic Resonance Imaging
Source: PLoS One. 2013 Jun 18;8(6):e65739. doi: 10.1371/journal.pone.0065739 (PMC3688799; doi:10.1371/journal.pone.0065739)
Supplement: Table S3 — Changes in frequency of the presence of glucose fluid in the jejunum, ileum, and ileum terminal during OGTT 3 months after surgery. OGTT: oral glucose tolerance test. (DOC) [file pone.0065739.s004.doc]

**Table S3. Changes in frequency of the presence of glucose fluid in the jejunum, ileum, and ileum terminal during OGTT** 3 months after surgery

| **Parameters** | **Before surgery** | **3 months after surgery** |
| --- | --- | --- |
| Presence of glucose fluid in the jejunum at 15 min (%) | 33.3 | 100 |
| Presence of glucose fluid in the jejunum at 30 min (%) | 41.7 | 100 |
| Presence of glucose fluid in the ileum at 15 min (%) | 11.1 | 88.9 |
| Presence of glucose fluid in the ileum at 30 min (%) | 33.3 | 100 |
| Presence of glucose fluid in the ileum terminal at 15 min (%) | 0 | 88.9 |
| Presence of glucose fluid in the ileum terminal at 30 min (%) | 22.2 | 100 |

OGTT: oral glucose tolerance test.Gable 3.linaluidrunum00000000000000000000000000000000000000000000000000000000000000000000000000000000000000000000000000000000000
