# Supplementary material for: Enhanced Intestinal Motility during Oral Glucose Tolerance Test after Laparoscopic Sleeve Gastrectomy: Preliminary Results Using Cine Magnetic Resonance Imaging
Source: PLoS One. 2013 Jun 18;8(6):e65739. doi: 10.1371/journal.pone.0065739 (PMC3688799; doi:10.1371/journal.pone.0065739)
Supplement: Table S4 — Differences in contraction frequencies between collapsed and fluid-distended bowel loops during OGTT 3 months after surgery. OGTT: oral glucose tolerance test. Data are presented as mean ± standard deviation. (DOC) [file pone.0065739.s005.doc]

**Table S4. Differences in contraction frequencies between collapsed and fluid-distended bowel loops during OGTT 3 months after surgery**

| **Parameters** | **Collapsed** | **Fluid- distended** | ***p*** |
| --- | --- | --- | --- |
| Contraction of the jejunum loops (frequency/min) | 0.5 ± 1.0 | 6.1 ± 0.7 | 0.000 |
| Contraction of the ileum loops (frequency/min) | 1.4 ± 1.6 | 7.4 ± 1.7 | 0.000 |

OGTT: oral glucose tolerance test. Data are presented as mean ± standard deviation.
