# Supplementary material for: Enhanced Intestinal Motility during Oral Glucose Tolerance Test after Laparoscopic Sleeve Gastrectomy: Preliminary Results Using Cine Magnetic Resonance Imaging
Source: PLoS One. 2013 Jun 18;8(6):e65739. doi: 10.1371/journal.pone.0065739 (PMC3688799; doi:10.1371/journal.pone.0065739)
Supplement: Table S5 — Representative results of intestinal motility during OGTT in 4 patients assessed by cine MRI. T2DM: type 2 diabetes mellitus; OGTT: oral glucose tolerance test; MRI: magnetic resonance imaging. (DOC) [file pone.0065739.s006.doc]

**Table S5. Representative results of intestinal motility during OGTT** in 4 patients assessed by cine MRI

|  | **OGTT before surgery** | | **OGTT after surgery** | |
| --- | --- | --- | --- | --- |
|  | **0 min** | **15 min** | **0 min** | **15 min** |
| **Patient #1** | **Slow** | **Slow** | **Slow** | **Accelerated** |
| (T2DM) | (Video S1A) | (Video S1B) | (Video S1C) | (Video S1D) |
| **Patient #2** | **Slow** | **Slow** | **Slow** | **Accelerated** |
| (T2DM) | (Video S2A) | (Video S2B) | (Video S2C) | (Video S2D) |
| **Patient #3** | **Slow** | **Slow** | **Slow** | **Accelerated** |
| (non-T2DM) | (Video S3A) | (Video S3B) | (Video S3C) | (Video S3D) |
| **Patient #4** | **Slow** | **Slow** | **Slow** | **Accelerated** |
| (non-T2DM) | (Video S4A) | (Video S4B) | (Video S4C) | (Video S4D) |

T2DM: type 2 diabetes mellitus; OGTT: oral glucose tolerance test; MRI: magnetic resonance imaging
